# Morning Exercise Reduces Abdominal Fat and Blood Pressure in Women; Evening Exercise Increases Muscular Performance in Women and Lowers Blood Pressure in Men

**DOI:** 10.3389/fphys.2022.893783

**Published:** 2022-05-31

**Authors:** Paul J. Arciero, Stephen J. Ives, Alex E. Mohr, Nathaniel Robinson, Daniela Escudero, Jake Robinson, Kayla Rose, Olivia Minicucci, Gabriel O’Brien, Kathryn Curran, Vincent J. Miller, Feng He, Chelsea Norton, Maia Paul, Caitlin Sheridan, Sheriden Beard, Jessica Centore, Monique Dudar, Katy Ehnstrom, Dakembay Hoyte, Heather Mak, Aaliyah Yarde

**Affiliations:** ^1^ Human Nutrition and Metabolism Laboratory, Department of Health and Human Physiological Sciences, Skidmore College, Saratoga Springs, NY, United States; ^2^ College of Health Solutions, Arizona State University, Phoenix, AZ, United States; ^3^ Department of Kinesiology, California State University, Chico, CA, United States

**Keywords:** cardiometabolic health, exercise time of day, abdominal fat, circadian rhythyms, muscular strength and power

## Abstract

The ideal exercise time of day (ETOD) remains elusive regarding simultaneous effects on health and performance outcomes, especially in women.

**Purpose:** Given known sex differences in response to exercise training, this study quantified health and performance outcomes in separate cohorts of women and men adhering to different ETOD.

**Methods:** Thirty exercise-trained women (BMI = 24 ± 3 kg/m^2^; 42 ± 8 years) and twenty-six men (BMI = 25.5 ± 3 kg/m^2^; 45 ± 8 years) were randomized to multimodal ETOD in the morning (0600–0800 h, AM) or evening (1830–2030 h, PM) for 12 weeks and analyzed as separate cohorts. Baseline (week 0) and post (week 12) muscular strength (1-RM bench/leg press), endurance (sit-ups/push-ups) and power (squat jumps, SJ; bench throws, BT), body composition (iDXA; fat mass, FM; abdominal fat, Abfat), systolic/diastolic blood pressure (BP), respiratory exchange ratio (RER), profile of mood states (POMS), and dietary intake were assessed.

**Results:** Twenty-seven women and twenty men completed the 12-week intervention. No differences at baseline existed between groups (AM vs PM) for both women and men cohorts. In women, significant interactions (*p* < 0.05) existed for 1RM bench (8 ± 2 vs 12 ± 2, ∆kg), pushups (9 ± 1 vs 13 ± 2, ∆reps), BT (10 ± 6 vs 45 ± 28, ∆watts), SJ (135 ± 6 vs 39 ± 8, ∆watts), fat mass (−1.0 ± 0.2 vs −0.3 ± 0.2, ∆kg), Abfat (−2.6 ± 0.3 vs −0.9 ± 0.5, ∆kg), diastolic (−10 ± 1 vs−5 ± 5, ∆mmHg) and systolic (−12.5 ± 2.7 vs 2.3 ± 3, mmHg) BP, AM vs PM, respectively. In men, significant interactions (*p* < 0.05) existed for systolic BP (−3.5 ± 2.6 vs −14.9 ± 5.1, ∆mmHg), RER (−0.01 ± 0.01 vs −0.06 ± 0.01, ∆VCO_2_/VO_2_), and fatigue (−0.8 ± 2 vs −5.9 ± 2, ∆mm), AM vs PM, respectively. Macronutrient intake was similar among AM and PM groups.

**Conclusion:** Morning exercise (AM) reduced abdominal fat and blood pressure and evening exercise (PM) enhanced muscular performance in the women cohort. In the men cohort, PM increased fat oxidation and reduced systolic BP and fatigue. Thus, ETOD may be important to optimize individual exercise-induced health and performance outcomes in physically active individuals and may be independent of macronutrient intake.

## Introduction

Humans exhibit circadian rhythms and diurnal variations in their physiology and in the time of day in which exercise is performed, typically falling into a morning or evening preferring chronotype (Aoyama and Shibata, 2020; Blazer et al., 2020). Expression of purported molecular clock genes exhibits an individualized diurnal variation, which correlates with muscular strength exercise performance and the clock itself (Kemler et al., 2020; Basti et al., 2021). The timing of exercise remains a controversial topic, with some investigators favoring morning exercise to enhance muscle adaptations and fuel utilization (Bennard and Doucet, 2006; Van Proeyen et al., 2010; Gonzalez et al., 2013; Ezagouri et al., 2019; Sato et al., 2019; Willis et al., 2020); whereas, others have shown afternoon/evening exercise is most favorable to improve muscle function (Bernard et al., 1998; Racinais, 2010; Chtourou and Souissi, 2012; Küüsmaa et al., 2016). In either case, research exploring potential effects of exercise time of day (ETOD) on training-induced adaptations remains to be fully chartered within multiple domains of “real-life” applicability, warranting examination. For instance, data is scarce regarding multimodal exercise regimens, psychological response, and potential differences in physiological response by ETOD (Teo et al., 2020; Teo et al., 2021), and nearly non-existent for healthy, exercise-trained individuals. Moreover, tightly controlled nutritional intake with adequate protein to promote exercise recovery and adaptation is often absent in ETOD interventions.

Over the last decade, increasing attention has focused on healthy lifestyle routines that are multimodal (i.e., resistance, high intensity intervals, stretching/flexibility, and endurance training) (Garber et al., 2011). Indeed, prior research has demonstrated that training programs which combine resistance and endurance exercises are more effective in improving body composition than either training modality alone (Beckers et al., 2008; Sillanpää et al., 2008; Marzolini et al., 2012; Lee et al., 2015). In addition to traditional resistance and endurance exercise, other routines are gaining in popularity, including stretching exercises such as yoga, tai chi, Pilates, and interval sprint training (Thompson, 2019). The American College of Sports Medicine (ACSM) has historically recommended moderate intensity physical aerobic activity but also includes resistance, flexibility, and neuromotor training exercises (Garber et al., 2011), though more recent guidelines suggest that inclusion of higher intensity interval training may result in more favorable cardiometabolic adaptations than traditional moderate-intensity aerobic training protocols (Campbell et al., 2019). Multimodal exercise has apparent benefits for athletes and recreational exercisers alike (Falk Neto and Kennedy, 2019). Moreover, such exercise regimens, encompassing multiple energy systems, movement patterns, and athletic range also have support under an evolutionary lens (Longman et al., 2020). Yet, recent research examining ETOD has focused on just one mode or a combination of resistance and endurance (aerobic) exercise regimens (Küüsmaa-Schildt et al., 2017; Blazer et al., 2021; Creasy et al., 2022; Souissi et al., 2022). Therefore, the current body of ETOD literature lacks critical data on the typical manner in which most athletes and fitness enthusiasts engage in physical activity programs. To this end, Arciero and colleagues have developed a systematic holistic approach to fitness training that includes a true multimodal approach of resistance functional exercise (“R”), interval sprint exercise (“I”), stretching (yoga, Pilates, tai chi; “S”) and endurance (“E”) exercise, collectively “RISE” training, and when combined with timed “protein pacing” intake (“PRISE”) results in significant improvements in fitness-related parameters, as well as enhanced cardiometabolic and body composition health (Arciero et al., 2014; Arciero et al., 2015; Arciero et al., 2016a; Arciero et al., 2016b; Ives et al., 2017). The RISE protocol is a multimodal exercise program with documented efficacy in both men and women, that may be an optimal way of meeting the current recommendations including the recent incorporation of high intensity interval exercise, with a tremendous amount of time-savings and feasible practicality.

Relative to men, women still remain generally under-studied in exercise science and sports medicine research with only ∼36% of study populations being women (Costello et al., 2014), if they are included at all. Some estimates suggest inclusion of women in sports science research to be as low as 3% (Brookshire, 2016). In addition, it’s well accepted that sex is a factor influencing acute and chronic training adaptations (Ansdell et al., 2020), as well as muscle function (Beaven et al., 2014), capillary density (Robbins et al., 2009), hunger responses (Tobin et al., 2021), and fat metabolism (Power and Schulkin, 2008) and therefore, we chose to analyze women and men as two separate cohorts in this study. Given the paucity of scientific investigations examining potential differences with time of day (TOD) in which training is performed on physical performance and health-related outcomes in healthy active women and the inherent physiologic and athletic performance differences compared to men, the primary aim of the present study was to examine the separate effects of multimodal RISE ETOD on fitness–and health-related performance outcomes in both healthy, normal weight women and men consuming a range of adequate protein intake (1.1–2.2 g/kg body weight). We hypothesized that the multimodal RISE training would improve fitness-related performance and cardiometabolic and body composition health outcomes, and that ETOD would have differential effects on body composition, physical performance, and health outcomes in women and men. Specifically, AM-exercise may result in greater improvements in cardiometabolic and body composition health, whereas, PM-exercise will foster greater improvements in muscle function and performance, with both AM and PM ETOD enhancing psychological mood state in men and women.

## Materials and Methods

### Participants

**FIGURE 1:**
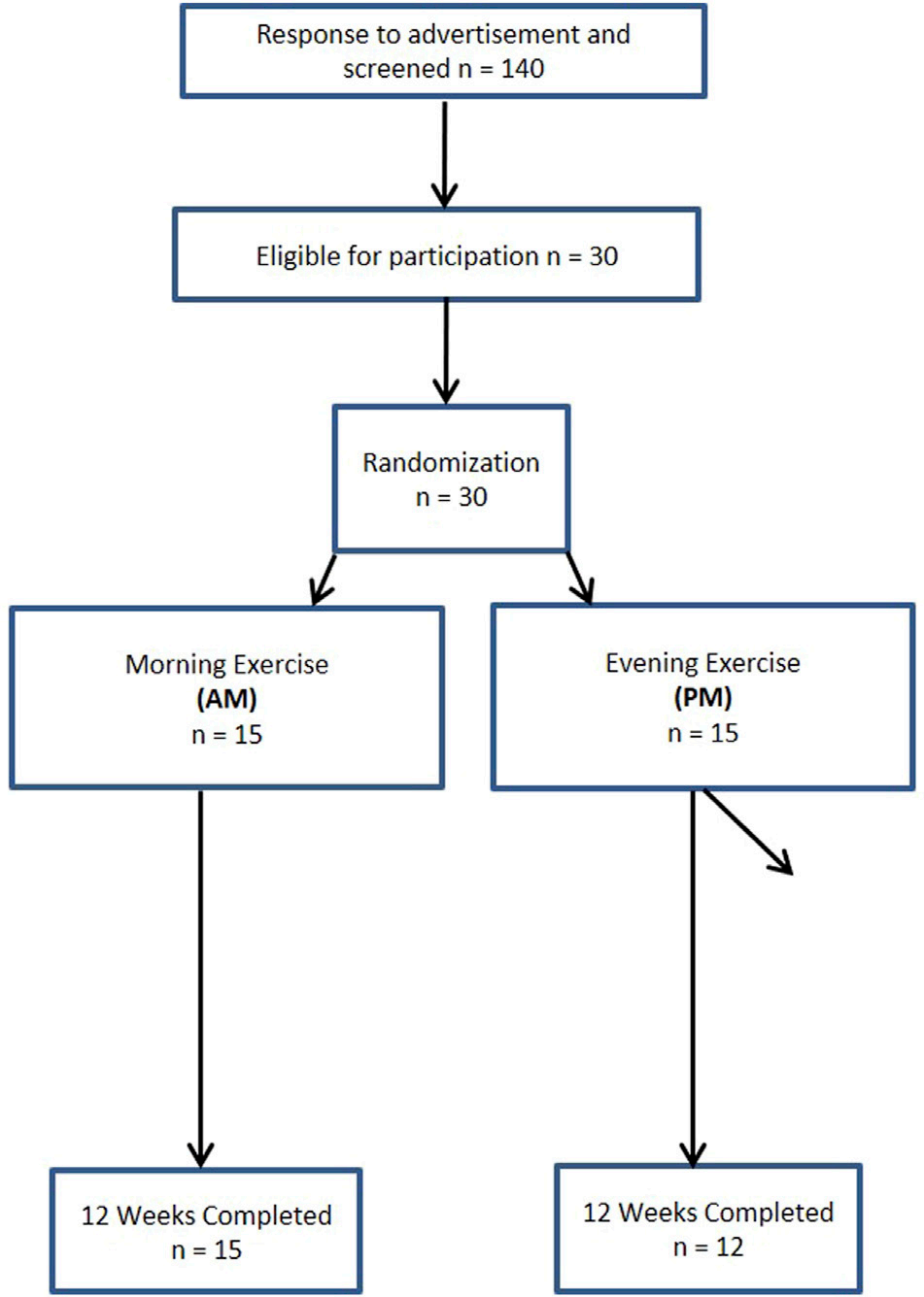
Consort experimental design for women.

**FIGURE 2:**
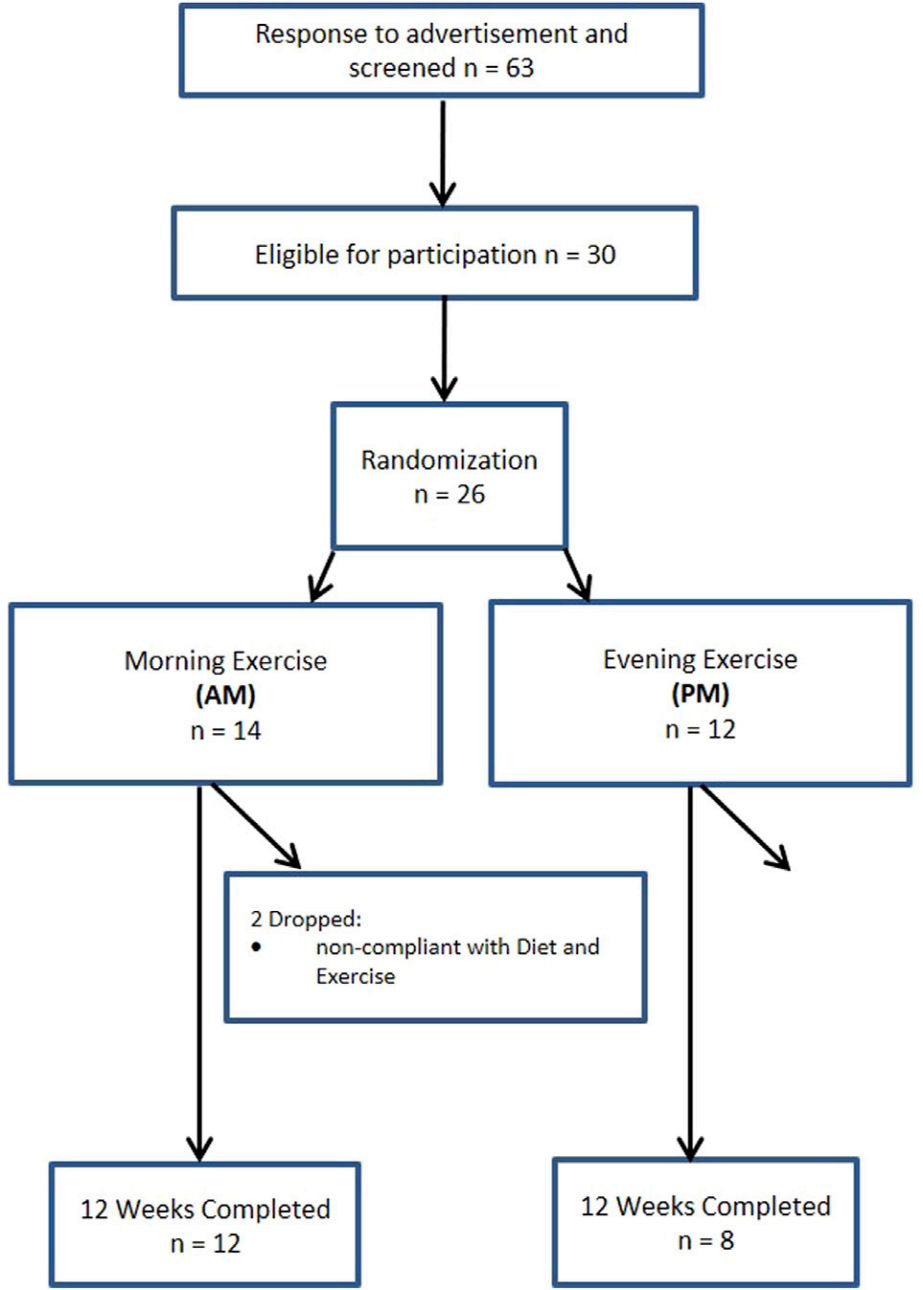
Consort experimental design for men.

A total of 140 women and 63 men from the Saratoga Springs, NY area, responded through emails, flyers and local newspapers to advertisements regarding the study. A total of 59 women and 30 men were initially screened, of which 30 females and 26 males were eligible for participation (Figures 1, 2 (Schulz et al., 2010)). Participants were nonsmoking, healthy, trained women and men with no known cardiovascular or metabolic diseases as assessed by a medical history and a comprehensive medical examination. All participants were highly active (>30 min, 4 days/week of structured physical activity, > 3 years), lean (BMI <25 kg/m2; % body fat <30%), middle aged (25–55 years), and weight stable (±2 kg) for at least 6 months prior to the beginning of the study. All participants provided informed written consent prior to participation, and the study was approved by the Human Subjects Institutional Review Board of Skidmore College (IRB #: 1401-382). All experimental procedures were performed in accordance with the Federal Wide Assurance and related New York State regulations, which are consistent with the National Commission for the Protection of Human Subjects of Biomedical and Behavioral Research and in agreement with the Helsinki Declaration as revised in 1983. This study was registered with ClinicalTrials.gov Identifier: NCT02593656.

### Experimental Design

#### Study Timeline

Participants were matched for body weight, body mass index (BMI) and percent body fat (%BF) and then randomly assigned to one of two groups: 1) resistance, interval, stretching, endurance (RISE) training performed in the morning (women AM, *n* = 15; men AM, *n* = 14); or 2) RISE exercise training performed in the evening (women PM, *n* = 15; men PM, *n* = 12) (Figures 1, 2). Women currently using oral contraceptives (*n* = 7; 4 a.m., 3 p.m.) were instructed to continue throughout the duration of the intervention. There were 8 peri/post-menopausal women included in the intervention (4 a.m.; 4 p.m.), all others (*n* = 19) reported normal menses. Women were not testing during the same menstrual phase, as this was not feasible given the number of study participants, laboratory and physical performance testing schedule conflicts, intensive training intervention, and funding, nor is this recommended by all investigators (Stanhewicz and Wong, 2020). All participants followed a supervised program of progressive exercise training for 12 weeks and were assigned either early morning exercise sessions (6:30-8:30a.m., AM) or evening sessions (6–8pm, PM). All testing procedures (see below) were administered pre-intervention (week 0) and post intervention (week 13) unless noted otherwise. Upon arrival at the laboratory, anthropometric and body composition measurements and blood sampling for subsequent analysis were performed.

### Nutritional Intervention, Meal Timing, and Dietary Assessment

The specific comparisons of the effects of varying macronutrient distribution (higher vs lower protein intake), including macronutrient distribution and meal timing protocols within women and men have been previously published (Arciero et al., 2016b; Ives et al., 2017) and therefore, only pertinent details regarding exercise time of day comparisons will be presented in this manuscript. Briefly, women and men participants in both groups were provided meal plans designed by a registered dietitian and instructed to follow the meal plans throughout the 12-week intervention. The registered dietitian met with participants weekly for the first two weeks and thereafter as needed. Participants followed a healthy meal plan providing adequate protein (1.0–2.0 g/kg body weight). Both AM and PM groups were provided equivalent nutritional support, as well as total energy and macronutrient distributions throughout the 12-week intervention.

The timing of meals was an important component of the current study. On resistance (R) and interval (I) exercise training days, participants consumed a small snack (∼250–300 kcals) < 1 h prior and on stretching (S) and endurance (E) training days arrived fasted (AM, overnight; PM, > 4 h) but well-hydrated and were allowed to consume water/electrolyte beverages as needed. Participants in AM exercise, consumed breakfast after their exercise routine and remaining meals were consumed at approximately 4-h intervals throughout the remainder of the day. For PM exercise participants, starting with breakfast and every 4 h, three meals were consumed prior to their exercise routines with the fourth meal consumed within an hour of finishing their evening exercise. On non-exercise days, participants consumed breakfast within an hour of waking in the morning and remaining meals were consumed every 4 h thereafter, with the final evening meal consumed within 2 h of going to bed.

Participants recorded daily food and beverage consumption during the 12-week intervention and analyzed from a representative 3-day period during weeks 0 and 12 to verify compliance (Food Processor SQL Edition, version 10.12.0, 2012; ESHA Research, Salem, OR) (Arciero et al., 2016b). The same research team member analyzed all diet records to minimize inter-subject variability.

### RISE Exercise Training Protocol

Women and men in both AM and PM groups underwent the same multi-modal exercise training regimen (RISE) with the same relative training volume, as described previously (Arciero et al., 2014; Arciero et al., 2016b; Ives et al., 2017). The training program consisted of four specific types of exercise training: 1) resistance exercise (R); 2) interval sprints (I); 3) stretching/yoga/Pilates (S); and 4) endurance exercise (E) (RISE training). Participants completed each of the four difference exercise routines one day per week for a total of four training sessions per week, each lasting less than an hour, with the exception of the endurance training bout, which was performed for 60 min or longer. All training sessions were performed in the Skidmore College Sports Center and supervised by a member of the research team to ensure proper technique, safety, and compliance with each of the four modes of training.

As a matter of reference, the resistance (R) training routine was comprised of a dynamic warm-up, lower and upper body resistance, followed by core muscle movements performed for 10-15 repetitions and for two to three sets, in order to induce muscular fatigue. A 30–60 s recovery was allowed between sets of the same exercise and between different types of exercises. The sprint interval (I) training sessions were comprised of 7–10 sets of 30–60 s of near maximal all-out sprints with 2–4 min of recovery/rest in between sets, such that the entire routine was completed within 35 min. Participants were free to choose any mode of exercise (treadmill, elliptical, stationary bike, swimming, snowshoeing, cycling, rollerblading, etc.) to complete the sprints. The stretching exercise routine were led by a certified yoga instructor and utilized a combination of familiar stretching and traditional yoga poses, as well as several additional Pilates movements such that all the major muscles and joints of the body were involved. Lastly, endurance exercise training included full body, rhythmical aerobic activities such as running, cycling, rowing, swimming, ellipticals, etc. and was performed for >60 min at 60% of maximal effort based on the heart rate reserve formula [((maximum heart rate−resting heart rate) × 0.6) + resting heart rate] via monitoring heart rate (Polar H7, Polar Electro, Lake Success, NY, United States) at every session to ensure subject safety and compliance with the exercise program.

### Laboratory Testing Procedures

Each laboratory testing day (weeks 0 and 13), for the measurement of anthropometric, body composition, and blood collection was performed between 0600 and 0900, following a 12-h fast and 48-h removed from caffeine and alcohol intake, and 48–72 h after the last exercise session. All laboratory testing day procedures have been previously published (Arciero et al., 2016b; Ives et al., 2017), and therefore only necessary details will be provided in the current manuscript.

### Physical Performance Assessments

All physical performance measures were obtained at the same time of day and completed over a two-day period at baseline (week 0) and post intervention (week 13) within 2 h of the same time of day exercise training was performed during the previous 12 weeks for AM and PM exercise groups, respectively. Specifically, aerobic power, muscular endurance, flexibility, and balance were completed on day one, while, upper and lower body strength and power tests and vertical jump were completed on day two. All baseline physical performance assessments were conducted following two familiarization sessions with a member of the research team to demonstrate proper technique and form for all tests prior to the actual testing days.

Upper and Lower Body Maximal Strength, Force and Power: One repetition maximum strength (1RM) on the bench and leg press using a barbell was performed pre and post intervention as previously described (Arciero et al., 2016b). One repetition maximum (1-RM) bench and leg press test-retest (r) and coefficient of variation (CV) from our laboratory with *n* = 15 are r = 0.99, CV = 1.6% and r = 0.99, CV = 2.7%, respectively. Within 15 min of completing the 1RM’s of the bench and leg press, participants performed dynamic bench throws (BT) and jump squats (JS), respectively using the Ballistic Measurement System (Innervations Inc., Muncie, IN) that is interfaced with a commercial-grade max smith rack. Prior to performing the tests participants performed 3-5 un-weighted practice trials for each movement. Following the warm-up, participants performed three consecutive repetitions with the barbell loaded to 30% of their predetermined IRM for SJ and 20% of their 1RM for BT (Arciero et al., 2016b; Ives et al., 2017). Spotters were located on either side of the barbell to ensure safety, as well as verbal encouragement.

Maximal vertical jump and reach was determined with the Vertec (Sports Imports, Columbus, OH) which consisted of two practice trials immediately before performing three vertical jump tests, that included a 30 s recovery in between each successive trial. The maximal jump height was calculated as the highest point reached and then subtracting out the participants’ maximal standing single-arm reach height.

Upper Body and Core Muscular Endurance: Upper body and core muscular endurance were assessed with timed push-ups and sit-ups in 1 minute, respectively (Arciero et al., 2016b; Ives et al., 2017). Bending the elbows to 90° followed by a return to the starting plank position (women started in plank position on the knees), was defined as a successful push-up. Timed sit-ups were considered successful if participants were able to complete a full curl up to a 90° position (vertical) to the floor and then return to the starting position with knees bent to 90 and back flat on the floor.

Standing Balance and Sit and Reach Flexibility: The stork balance test was used to assess postural standing balance with participants standing on their dominant leg while the heel was lifted off the ground and the non-dominant leg is bent 90° with the sole of the foot placed along the inside of the dominant knee (Arciero et al., 2016b; Ives et al., 2017). Three criteria were used to determine the end of the trial; 1) the heel of the dominant leg touched the ground, 2) the hands came off of the hips, or 3) the non-dominant foot was removed from the dominant standing leg and the best of three attempts was used as the final score. Flexibility of the lower back and hamstrings was assessed with the sit and reach test using a standard sit and reach box (Lafayette Instrument Company, Lafayette, IN). The maximal distance reached of 3 trials was recorded.

Aerobic Power 5 Km Cycle Ergometer Time Trial: All participants completed a 5-km time trial (5 km TT) on the Velotron Dynafit Pro cycle ergometer (Racermate, CompuTrainer 3D Software, Version 1, Seattle, WA, United States) at weeks 0 and 13. The TT began with a 5–7 min warm-up followed by a self-selected pedaling cadence and gear ratio to complete the trial as fast as possible. Total time, mean, and maximum watts, heart rate and blood pressure were continuously recorded throughout the trial.

### Total and Regional Body Composition

Body weight was measured using a digital scale (Befour Inc., Cedarburg, WI 53080 model number FS0900) and waist circumferences (cm) were measured at the area two cm above the iliac crest using a standard tape measure. Total and regional body composition was obtained with Dual Energy X-Ray Absorptiometry (iDXA; Lunar iDXA; GE Healthcare, Madison, WI; using encore software version 18) (Arciero et al., 2016b). All women were required to sign a waiver stating they were not currently, or planning on being, pregnant during the study. Test-retest intraclass correlation (r) and coefficient of variation (CV) for body composition analysis using iDXA in our laboratory with *n* = 12 is: Fat-free mass, fat mass (kg) and abdominal fat mass (%) intraclass correlations (r) and coefficient of variations (CVs) was r = 0.99, CV = 0.64%, r = 0.98, CV = 2.2%, and r = 0.99, CV = 2.4%, respectively.

### Cardiometabolic Biomarkers

Blood Biomarkers: A 20 ml blood sample was obtained from the antecubital vein of the forearm at weeks 0 and 13 and collected into vacutainer tubes (EDTA-coated), centrifuged (Hettich Rotina 46R5) for 15 min at 2,500 rpm at 4°C and aliquoted into cryovials for storage at −70°C until analyzed. Insulin was measured using ELISA kits (Millipore, Billerica MA) and glucose, total cholesterol (TC), low-density lipoprotein cholesterol (LDL-C), and triglycerides (TRG) were assessed using the Cholestech LDX blood analysis system (Hayward, CA). Plasma C-reactive protein, insulin, IL-6, and cortisol concentrations were determined using commercially available ELISA kits (Millipore, Billerica MA). Total cholesterol and low-density lipoprotein cholesterol (mg/dl) test-retest intraclass correlation (r) and coefficient of variation (CV) from *n* = 15 in our laboratory is r = 0.95, CV = 3.2%, and r = 0.97, CV = 5.3%, respectively.

Blood Pressure and Heart Rate: Blood pressure and heart rate were obtained with a single measurement using an automated blood pressure monitor (Omron Healthcare Inc., Milton Keynes, United Kingdom) following >15 min of quiet sitting.

Vascular Stiffness: Arterial stiffness was quantified from augmentation index (Aix) *via* pulse contour analysis and pulse wave velocity using arteriography (Arteriograph, version 1.10.0.1, TensioMed Kft., Budapest, Hungary) (Arciero et al., 2014; Arciero et al., 2016a). Similarly, the aortic pulse wave velocity (PWVao) was calculated from the wave reflection amplitude generated from the back flow of the pulse wave from the aortic bifurcation. Return time (RT) was calculated from the time interval between peaks from the early direct (P_1_) and reflected late (P_2_) systolic waves and were derived from measurements of the distance from the upper edge of the pubic bone to the sternal notch (Jugulum-Symphisis¼).

Resting Metabolic Rate (RMR) & Respiratory Exchange Ratio (RER) and Substrate Utilization: Indirect calorimetry with the ventilated hood was used to measure resting metabolic rate (RMR) (ParvoMedic; True One 2,400 software, Salt Lake City UT, United States) between 0600 and 0800 (Arciero et al., 2016b; Ives et al., 2017). The RER and fat and carbohydrate substrate utilization rates were calculated from gas exchange (indirect calorimetry) using standardized caloric equivalents, as the volume of carbon dioxide produced to the volume of oxygen consumed (VCO_2_/VO_2_). RMR (kcal/min) intraclass correlation (r) and coefficient of variation (CV) from our laboratory with n = 14 is r = 0.92, 4.2%, respectively.

### Behavioral Mood State, and Feelings of Hunger and Satiety

Psychological mood state changes over the course of the study were assessed with the Profile of Mood States questionnaire (POMS) at the beginning (week 0) and end (week 13) of the study to quantify changes in six major mood states; tension, depression, anger, vigor, fatigue, and confusion. Total mood disturbance (TMD) was also calculated using the following formula:

TMD=(tension + depression + anger + fatigue + confusion)-vigor

Feelings of hunger, satiation, and desire-to-eat were assessed with visual analog scales (VAS, 0–100 mm) at baseline (week 0) and week 13 to evaluate the impact of the lifestyle interventions (Arciero et al., 2016b).

### Statistical Analysis

Women and men were analyzed as separate cohorts given the inherent sex differences in response to exercise training, as previously discussed (see Introduction). A two way (2 × 2) repeated measures ANOVA was performed to assess the main effects of time (baseline, week 0 vs post-intervention, week 13) and interactions of group (AM vs PM, exercise time of day, ETOD) by time (weeks 0 vs 13). No adjustments were made for multiple comparisonsPost hoc comparisons (Bonferroni) were performed if there was a significant interaction or main effects. All values are reported as means ± SD unless stated otherwise and all statistical analyses were performed using SPSS software (Ver. 23; IBM, Armonk, NY). Significance was set at *p* < 0.05 using a one-tailed approach based on the directional hypotheses stated above. Post hoc power analysis revealed that n = 10 study participants were needed to detect statistical significance (*p* < 0.05) between AM vs PM ETOD within both groups of women and men. Normality statistics (Shapiro–Wilk’s tests and skewness and kurtosis z-scores) and probability plots (Q-Q plots and histograms) were generated to test normality assumptions, and log transformations were performed as appropriate.

## Results

### Baseline Participant Characteristics

Participant characteristics for both women and men groups are presented in Table 1. Prior to the intervention, participants were well matched between groups (AM vs PM) for nearly all characteristics in each outcome domain (physical performance, cardiometabolic health, body composition, and diet).

**TABLE 1:** Baseline characteristics between exercise time of day groups (AM vs PM) for women and men.

	Women	
	AM	PM
	(*n* = 14)	(*n* = 13)
Age (yr)	42 ± 7	42 ± 9
Height (cm)	165 ± 7	166 ± 6
Weight (kg)	63 ± 8	67 ± 8
Body mass index (kg/m^2^)	23 ± 2	24 ± 3
Heart rate (beats/min)	61 ± 9	58 ± 9
Systolic blood pressure (mmHg)	131 ± 15	120 ± 7^a^
Diastolic blood pressure (mmHg)	83 ± 11	75 ± 5
	**Men**	
	**AM (*n* = 12)**	**PM (*n* = 8)**
Age (yr)	44 ± 7	47 ± 9
Height (cm)	181 ± 9	177 ± 9
Weight (kg)	82 ± 15	82 ± 7
Body mass index (kg/m^2^)	25 ± 3	26 ± 3
Heart rate (beats/min)	54 ± 7	57 ± 4
Systolic blood pressure (mmHg)	116 ± 8	124 ± 6^a^
Diastolic blood pressure (mmHg)	74 ± 8	76 ± 4

a *p* < 0.05 AM vs PM., Data displayed as means ± standard deviation.

*Women* There were no differences within the women cohort for AM and PM groups prior to the start of the intervention, with the exception of systolic blood pressure (*p* < 0.05). Three women participants in PM group were excluded due to non-compliance with the diet and/or exercise routine, resulting in an overall 90% adherence rate, and the non-compliant were removed from all subsequent analysis.

*Men*. Similarly, no differences existed within the men cohort with the exception of systolic blood pressure (*p* < 0.05). Four men participants in the PM and two in the AM group were excluded due to non-compliance, resulting in a 77% adherence, and removed from final analysis.

### Impact of Exercise Time of Day on Dietary Intake

*Women*. Self-reported dietary intake for all macro- and micro-nutrients was unchanged during the 12-week exercise intervention for all variables and no differences existed between women in the AM and PM exercise groups (Table 2).

**TABLE 2:** Dietary intake changes between AM vs PM exercisers during the 12-week RISE Exercise Intervention for women and men.

	Pre-training		Post-training	
	AM	PM	AM	PM
Women				
Energy (kcals/day)	1,663 ± 232	1,610 ± 260	1,678 ± 235	1,685 ± 263
Fat (g/day)	60 ± 19	56 ± 12	57 ± 24	53 ± 13
Fat (%)	15 ± 5	14 ± 3	14 ± 5	13 ± 3
Carbohydrate (g/day)	172 ± 55	187 ± 69	181 ± 44	187 ± 56
Carbohydrate (%)	40 ± 10	47 ± 13	43 ± 9	47 ± 8
Protein (g/day)	72 ± 13	82 ± 18	92 ± 30	109 ± 46
Protein (g/kg BW/day)	1.1 ± 0.3	1.3 ± 0.3	1.5 ± 0.5	1.6 ± 0.6
Protein (%)	17 ± 4	20 ± 7	21 ± 6	25 ± 9
Sodium (mg/day)	1758 ± 600	1837 ± 937	1927 ± 565	1811 ± 700
Sugar (g/day)	68 ± 32	75 ± 33	72 ± 40	75 ± 21
Fiber (g/day)	21 ± 7	20 ± 8	24 ± 9	26 ± 10
Men				
Energy (kcals/day)	1974 ± 443	2,205 ± 286	2,140 ± 390	2,274 ± 164
Fat (g/day)	70 ± 35	69 ± 26	67 ± 18	77 ± 28
Fat (%)	14 ± 4	13 ± 3	13 ± 3	13 ± 4
Carbohydrate (g/day)	223 ± 37	201 ± 26	251 ± 61	222 ± 13
Carbohydrate (%)	46 ± 9	38 ± 8	46 ± 7	39 ± 1
Protein (g/day)	103 ± 32	135 ± 55	144 ± 52	143 ± 59
Protein (g/kg BW/day)	1.3 ± 0.4	1.6 ± 0.6	1.8 ± 0.7	1.7 ± 0.7
Protein (%)	21 ± 4	26 ± 7	26 ± 8	25 ± 8
Sodium (mg/day)	2,454 ± 1,331	2,621 ± 886	2,561 ± 1,317	2,574 ± 1,134
Sugar (g/day)	67 ± 23	71 ± 14	79 ± 30	89 ± 14
Fiber (g/day)	30 ± 13	26 ± 9	32 ± 11	22 ± 11

Data are means ± SD.

*Men*. Dietary intake for all macro- and micro-nutrients were unchanged throughout the 12-week intervention for men, as well and no differences were observed between the AM and PM exercisers.

### Impact of Exercise Time of Day on Training-Induced Changes in Physical Performance

**FIGURE 3:**
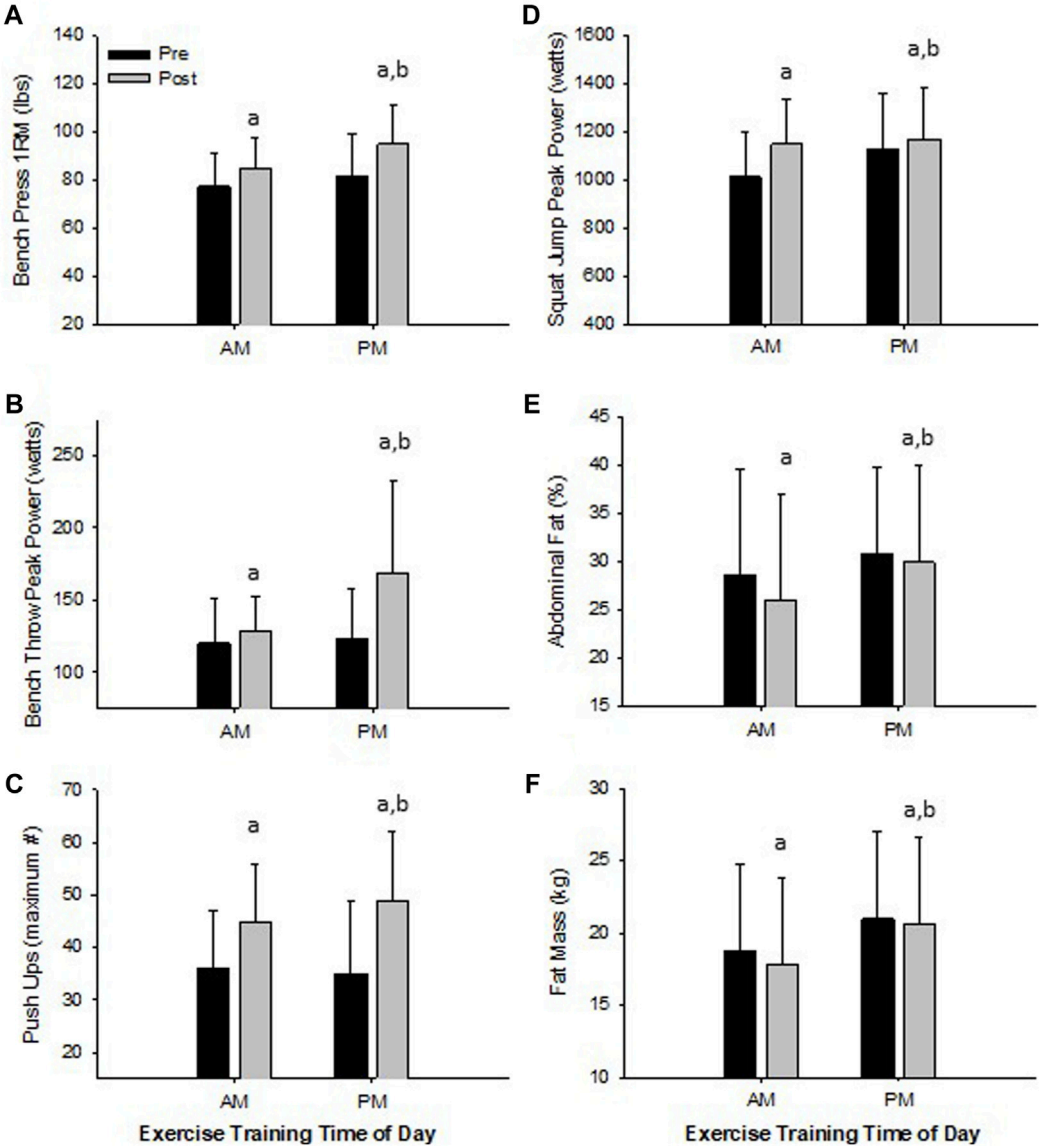
Significant Changes in Women with RISE Exercise Training Conducted in the Morning (AM) or Evening (PM). Upper Body Maximal Strength **(A)**, Upper Body Peak Power **(B)**, Upper Body Muscle Strength/Endurance **(C)**, Lower Body Peak Power **(D)**, Abdominal Fat Percent **(E)**, and Fat Mass **(F)**. ^a^ *p* < 0.05 pre vs post pairwise differences; ^b^ *p* < 0.05 group × time interaction. Data are means ± SD.

*Women*. All physical performance outcomes in women improved following the RISE exercise intervention in both AM and PM groups, including upper and lower body muscle function (*p* < 0.05, Table 3 and Figures 3A–D). A significant interaction existed for ETOD whereby PM-exercisers improved to a significantly greater extent on upper body maximal strength (bench press 1RM; *p* = 0.02, Figure 3A), peak power (bench throws; *p* = 0.01, Figure 3B), and muscular endurance (pushups; *p* = 0.04, Figure 3C), whereas, AM-exercisers improved more on lower body peak power (jump squats; *p* = 0.02, Figure 3D). Thus, the time of day in which women performed exercise training (AM vs PM), significantly altered the training-induced adaptation in upper body strength, power, muscular endurance and lower body muscular power (Table 3; Figures 3A–D), with PM-exercisers exhibiting greater improvements in upper body muscle function and AM-exercisers showing enhanced lower body peak power.

**TABLE 3:** Physical performance and body composition changes between AM vs PM exercisers during the 12-weeks RISE Exercise Intervention for women and men.

	Pre-training		Post-training	
	AM	PM	AM	PM
**Physical Performance**				
Women				
Bench press 1 RM (kg)	36 ± 6	37 ± 8	39 ± 6^a^	43 ± 7^a^ ^,^ ^b^
Bench throw peak power (watts)	120 ± 31	123 ± 35	129 ± 24^a^	169 ± 63^a^ ^,^ ^b^
Push-ups (reps)	36 ± 11	35 ± 14	45 ± 11^a^	49 ± 13^a^ ^,^ ^b^
Leg press 1 RM (kg)	121 ± 54	143 ± 59	176 ± 55^a^	200 ± 48^a^
Squat jump peak power (watts)	1,010 ±185	1,126 ± 229	1,145 ± 191^a^ ^,^ ^b^	1,165 ± 218^a^
Sit-ups (reps)	32 ± 8	33 ± 6	39 ± 7^a^	41 ± 8^a^
Sit and reach (cm)	37 ± 5	35 ± 10	40 ± 5^a^	39 ± 7^a^
5 km time trial (sec)	624 ± 52	611 ± 26	590 ± 35^a^	587 ± 29^a^
Men				
Bench press 1 RM (kg)	77 ± 18	75 ± 21	85 ± 17^a^	82 ± 17^a^
Bench throw peak power (watts)	381 ± 112	372 ± 121	419 ± 120^a^	423 ± 87^a^
Push-ups (reps)	41 ± 13	43 ± 15	50 ± 15^a^	54 ± 11^a^
Leg press 1 RM (kg)	207 ± 53	208 ± 78	270 ± 56^a^	261 ± 86^a^
Squat jump peak power (watts)	1836 ± 285	1804 ± 256	1936 ± 264^a^	1930 ± 309^a^
Sit-ups (reps)	35 ± 9	34 ± 6	40 ± 9^a^	46 ± 11^a^
Sit and reach (cm)	27 ± 10	23 ± 10	31 ± 10^a^	25 ± 10^a^
5 km time trial (sec)	556 ± 48	547 ± 72	527 ± 42^a^	528 ± 64^a^
**Body composition**				
Women				
Body fat (%)	30.7 ± 7.1	32.2 ± 5.6	29.3 ± 7.2^a^	31.4 ± 5.7^a^
Abdominal fat (%)	28.7 ± 10.7	30.9 ± 9.4	26.1 ± 11.0^a^ ^,^ ^b^	30.0 ± 10.0^a^
Hip fat (%)	35.3 ± 6.8	36.6 ± 4.8	33.3 ± 7.0^a^	35.3 ± 4.7^a^
Fat mass (kg)	18.8 ± 6.0	21.0 ± 5.6	17.8 ± 5.8^a^ ^,^ ^b^	20.6 ± 5.9^a^
Fat-free mass (%)	70.2 ± 7.1	68.6 ± 5.7	71.7 ± 7.2^a^	70.0 ± 6.2^a^
Men				
Body fat (%)	22.5 ± 6.6	24.9 ± 5.3	21.6 ± 6.5^a^	23.6 ± 5.4^a^
Abdominal fat (%)	26.7 ± 12.2	29.2 ± 8.8	25.1 ± 12.0^a^	27.2 ± 10.1^a^
Hip fat (%)	21.2 ± 5.6	25.1 ± 5.1	19.8 ± 5.6^a^	23.4 ± 4.2^a^
Fat mass (kg)	18.6 ± 8.9	19.5 ± 4.6	17.7 ± 8.1^a^	18.4 ± 5.0^a^
Fat-free mass (%)	78.1 ± 6.6	75.7 ± 5.2	79.5 ± 6.5^a^	77.2 ± 4.7^a^

a p < 0.05 main effect of time

b p < 0.05 interaction of ETOD and intervention.

Data are means ± SD.

*Men*. Similarly, in men, all physical performance outcomes significantly improved in both AM and PM groups following the RISE exercise training (Table 3). However, in contrast to the women, there were no differences between the AM and PM exercisers for any performance variable.

### Impact of Exercise Time of Day on Training-Induced Changes in Body Composition

*Women*. Both AM and PM ETOD groups significantly reduced total body fat (kg, %), abdominal and hip fat (%), and increased fat-free mass (%) following the RISE exercise training intervention in women (Table 3; Figure 3E,F). However, the magnitude of improvement was significantly greater in AM exercisers for total body fat mass (*p* = 0.04) and abdominal fat percentage (*p* = 0.03; Table 3; Figure 3E,F). It’s important to highlight these favorable body composition changes occurred in the absence of changes in body weight (Pre; AM 63 ± 8, PM 67 ± 8 kg: Post; AM 63 ± 8, PM 67 ± 8 kg, *p* > 0.05).

*Men*. Total body fat mass and body fat (%), abdominal and hip fat (%) decreased, and fat-free mass (%) increased significantly in AM and PM groups for men (Table 3), with no differences between groups. Similar to women, body weight did not change in either AM or PM groups (Pre; AM 82.4 ± 15.4, PM 81.7 ± 7.1 kg: Post; AM 81.8 ± 14.5, PM 80.6 ± 7.4 kg, *p* > 0.05).

### Impact of Exercise Time of Day on Training-Induced Changes on Cardiometabolic Outcomes

*Women*. Total cholesterol, systolic and diastolic blood pressure, aortic and brachial augmentation index decreased significantly in both AM and PM exercise groups (*p* < 0.05, Table 4). Moreover, AM exercisers reduced systolic and diastolic blood pressure to a greater degree than PM exercisers (*p* < 0.05, Table 4). Interestingly, resting metabolic rate decreased significantly (*p* < 0.05) only in AM (9.1%) versus PM (3.5%) exercisers following the RISE exercise intervention, whereas, respiratory exchange ratio remained unchanged.

**TABLE 4:** Cardiometabolic changes between AM vs PM exercisers during the 12-week RISE exercise intervention for women and men.

	Pre-training	Post-training		
	AM	PM	AM	PM
Women				
**Biochemistry**				
Total Cholesterol (mg/dl)	185 ± 35	185 ± 24	175 ± 22^a^	183 ± 25^a^
HDL Cholesterol (mg/dl)	66 ± 17	68 ± 13	67 ± 12	70 ± 13
LDL Cholesterol (mg/dl)	87 ± 14	104 ± 25	81 ± 17	102 ± 26
Triglycerides (mg/dl)	94 ± 41	88 ± 41	102 ± 29	79 ± 26
TC:HDL (ratio)	2.8 ± 0.8	2.8 ± 0.6	2.6 ± 0.4	2.7 ± 0.6
Glucose (mg/dl)	81 ± 5	81 ± 8	84 ± 5	83 ± 6
Insulin (µU/ml)	2.4 ± 0	2.7 ± 1	2.4 ± 1	2.5 ± 0
hsCRP (µU/ml)	0.7 ± 1	0.3 ± 1	0.7 ± 2	0.4 ± 1
IL-6 (pg/ml)	1.7 ± 4	2.8 ± 7	2.3 ± 5	5.1 ± 8
Cortisol (ng/ml)	95 ± 32	94 ± 21	85 ± 28	97 ± 19
**Vascular Health**				
Heart rate (bpm)	61 ± 10	58 ± 9	60 ± 8	56 ± 7
SBP (mmHg)	131 ± 15	120 ± 7	118 ± 13^a^ ^,^ ^b^	117 ± 11^a^
DBP (mmHg)	83 ± 11	75 ± 5	73 ± 9^a^ ^,^ ^b^	70 ± 11^a^
Aortic AIx (%)	36 ± 10	34 ± 11	34 ± 10^a^	33 ± 9^a^
Brachial AIx (%)	-2 ± 19	-6 ± 21	-7 ± 19^a^	-10 ± 17^a^
**Metabolism**				
RER (VCO2/VO2)	0.80 ± 0.06	0.80 ± 0.10	0.81 ± 0.05	0.78 ± 0.04
RMR (kcals/day)	1,438 ± 200	1,391 ± 148	1,307 ± 136^b^	1,342 ± 127
Men				
**Biochemistry**				
Total Cholesterol (mg/dl)	182 ± 24	162 ± 18	186 ± 25	158 ± 21
HDL Cholesterol (mg/dl)	51 ± 13	44 ± 13	53 ± 14	47 ± 10
LDL Cholesterol (mg/dl)	118 ± 21	101 ± 14	125 ± 15	97 ± 18
Triglycerides (mg/dl)	129 ± 46	92 ± 34	122 ± 60	79 ± 23
TC:HDL (ratio)	3.7 ± 0.3	3.8 ± 0.3	3.6 ± 0.3	3.4 ± 0.3^b^
Glucose (mg/dl)	84 ± 9	78 ± 4	86 ± 11	80 ± 5
Insulin (µU/ml)	2.7 ± 1	2.9 ± 1	2.7 ± 1	2.8 ± 1
hsCRP (µU/ml)	0.3 ± 0	0.6 ± 1	0.2 ± 0	1.0 ± 1
IL-6 (pg/ml)	3.3 ± 5	1.4 ± 1	3.3 ± 2	2.9 ± 3
Cortisol (ng/ml)	93 ± 16	102 ± 29	83 ± 25	108 ± 22
**Vascular Health**				
Heart rate (bpm)	53 ± 6	54 ± 2	51 ± 5^a^	50 ± 6^a^
SBP (mmHg)	116 ± 7	124 ± 6	113 ± 6	109 ± 10^b^
DBP (mmHg)	74 ± 8	76 ± 4	76 ± 6	72 ± 7
Aortic AIx (%)	33 ± 5	31 ± 6	30 ± 3	26 ± 4
Brachial AIx (%)	-10 ± 9	-13 ± 11	-15 ± 7	-23 ± 8
**Metabolism**				
RER (VCO2/VO2)	0.83 ± 0.02	0.85 ± 0.02	0.82 ± 0.01	0.80 ± 0.01^b^
RMR (kcals/day)	1876 ± 329	1720 ± 135	1945 ± 362	1,676 ± 319

HDL, High Density Lipoprotein; LDL, Low Density Lipoprotein; hsCRP, high sensitivity C-Reactive Protein; IL-6, Interleukin 6; SBP, systolic blood pressure; DBP, diastolic blood pressure; Aortic AIx, aortic augmentation index; Brachial AIx, brachial augmentation index; RER, respiratory exchange ratio.

a p < 0.05 main effect of time

b p < 0.05 interaction of ETOD and intervention.

Data are means ± standard deviation.

**FIGURE 4:**
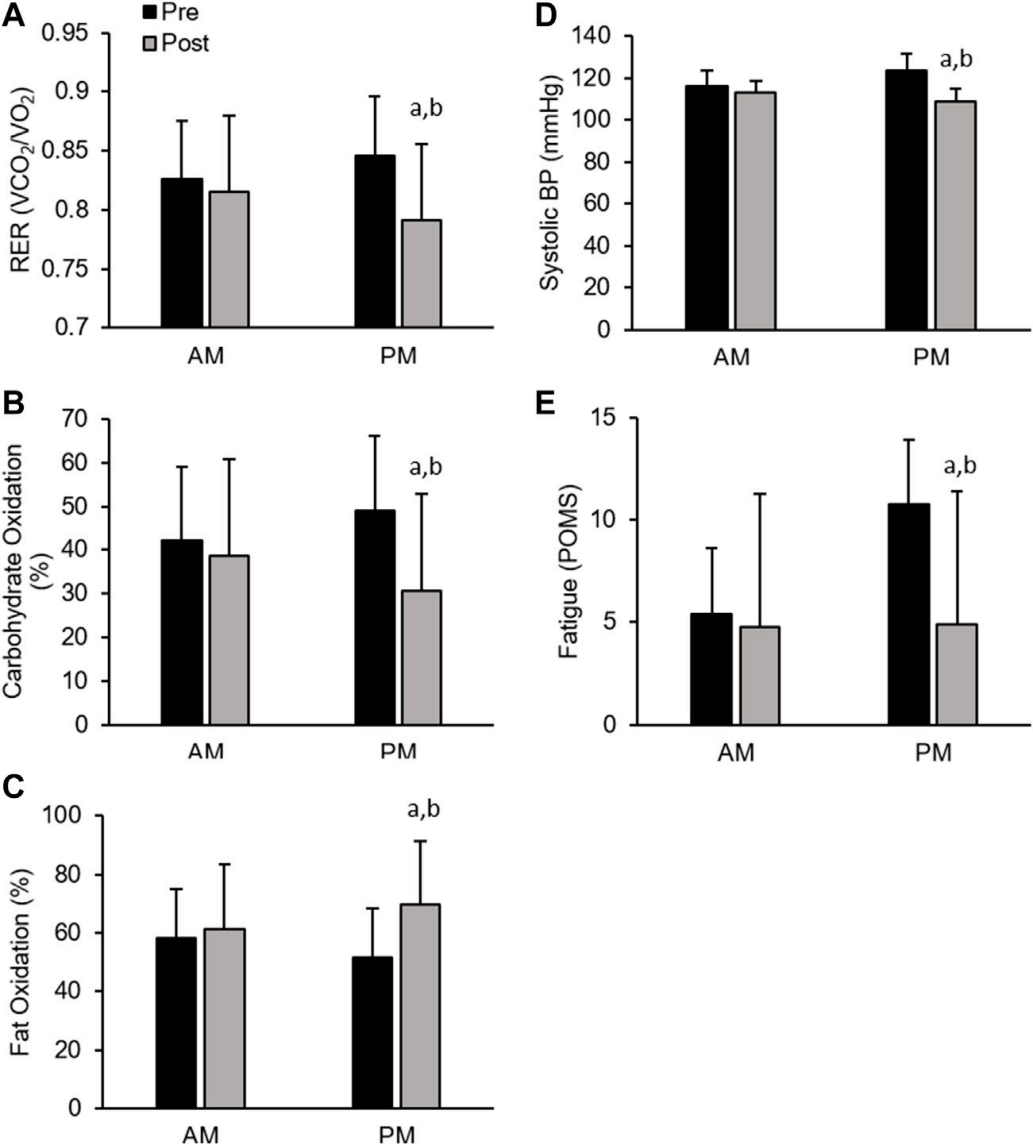
Significant Changes in Men with RISE Exercise Training Conducted in the Morning (AM) or Evening (PM). Respiratory Exchange Ratio (RER, **(A)**, Relative Carbohydrate Oxidation (%, **(B)**, and Relative Fat Oxidation (%, **(C)**, Systolic Blood Pressure **(D)**, Fatigue (POMS, **(E)**. ^a^ *p* < 0.05 pre vs post pairwise differences; ^b^ *p* < 0.05 group × time interaction. Data are means ± SD.

*Men*. All cardiometabolic variables were unchanged in AM exercisers, whereas, total cholesterol:HDL-cholesterol, systolic blood pressure (SBP) and respiratory exchange ratio (RER) declined significantly in PM compared to AM exercisers (*p* < 0.05, Table 4). Most notable was the significant reduction in RER in PM versus AM men exercisers, which mirrored a significant increase in fat oxidation (+28.4 ± 6.9% PM vs +9.1 ± 5.7% AM; *p* < 0.05) and decrease in carbohydrate oxidation (−37.5 ± 11.2% PM vs −8.1 ± 1.1% AM; *p* < 0.05) over the course of the 12-weeks study. All other cardiovascular and metabolic (glucose, insulin), and inflammatory (hsCRP, IL-6, cortisol) biomarker panels were unchanged in both groups (Table 4; Figures 4A–D).

### Impact of Exercise Time of Day on Training-Induced Changes in Mood, Satiety, and Hunger

*Women*. Behavioral mood state, assessed with the profile of mood states (POMS) questionnaire, showed an interaction effect for feelings of tension and total mood disturbance, and post hoc analysis confirmed this response occurred only in the PM exercisers (Table 5). In addition, the RISE exercise intervention demonstrated increased satiety in PM exercising women, whereas no changes occurred in mood or hunger ratings in AM exercisers.

**TABLE 5:** Profile of Mood States (POMS) and Visual Analog Scale (VAS) changes between AM vs PM exercisers during the 12-weeks RISE Exercise Intervention for women and men.

	Pre-training		Post-training	
	AM	PM	AM	PM
**POMS measures**				
Women				
Tension	6 ± 3	7 ± 8	5 ± 3	4 ± 4^b^
Depression	3 ± 2	4.± 8	2 ± 2	2 ± 4
Anger	4 ± 3	3 ± 2	3 ± 3	3 ± 4
Fatigue	5 ± 3	5 ± 6	5 ± 4	4 ± 7
Confusion	4 ± 2	4 ± 5	3 ± 2	3 ± 4
Vigor	20 ± 4	19 ± 4	20 ± 4	21 ± 7
Total Mood Disturbance	1 ± 10	5 ± 28	−2 ± 12	−4 ± 29^b^
Men				
Tension	10 ± 9	9 ± 4	6 ± 6^a^	5 ± 4^a^
Depression	8 ± 9	5 ± 3	2 ± 2^a^	3 ± 3^a^
Anger	10 ± 14	7 ± 4	4 ± 5^a^	4 ± 5^a^
Fatigue	5 ± 3	11 ± 7	5 ± 5^a^	5 ± 4^ab^
Confusion	5 ± 3	5 ± 3	3 ± 2^a^	4 ± 3^a^
Vigor	19 ± 7	18 ± 5	21 ± 8	18 ± 4
Total Mood Disturbance	20 ± 35	19 ± 17	−3 ± 20^a^	4 ± 12^a^
**VAS measures**				
Women				
Desire to eat (mm)	45 ± 23	39 ± 25	47 ± 19	43 ± 25
How full? (mm)	26 ± 15	26 ± 23	32 ± 17	38 ± 20^b^
How hungry? (mm)	41 ± 16	41 ± 22	46 ± 17	43 ± 22
How much food? (mm)	45 ± 15	48 ± 17	54 ± 15	44 ± 15^b^
Men				
Desire to eat (mm)	45 ± 31	45 ± 14	42 ± 28	41 ± 20
How full? (mm)	26 ± 19	34 ± 22	34 ± 19	28 ± 10
How hungry? (mm)	46 ± 26	47 ± 17	38 ± 29	36 ± 14
How much food? (mm)	54 ± 21	52 ± 16	59 ± 26	47 ± 12

a *p* < 0.05 main effect of time.

b *p* < 0.05 interaction of ETOD, and intervention. Data are means ± SD.

*Men*. In contrast, men showed significant improvement in most mood states, including tension, depression, anger, fatigue, confusion, and total mood disturbance regardless of ETOD. Interestingly, feelings of fatigue declined significantly more in PM compared to AM exercising men (Table 5; Figure 4E). All measures of satiety and hunger remained unchanged over the duration of the 12-week RISE exercise intervention in both ETOD groups.

Correlational analysis in the combined group of women revealed improved mood was strongly associated with lowered blood pressure response (r = - 0.37; *p* < 0.05), as well as greater gains in fat-free mass (r =−0.36; *p* < 0.05). Whereas, in men, correlation analysis showed significant relationships between reductions in overall mood state (TMD) and respiratory exchange ratio, fat mass, abdominal fat, and hip fat (r = 0.56; r = 0.50; r = 0.56; *r* = 0.73, respectively; *p* < 0.05), further supporting a link between improvements in mood with enhanced cardiometabolic and body composition outcomes.

## Discussion

This study provides novel insight into the impact of exercise time of day (ETOD) and associated circadian rhythms on physical performance, body composition, cardiometabolic health and psychological mood state outcomes in separate cohorts of healthy, exercise-trained women and men performing exercise either early in the morning (6:00–8:00AM) or later in the evening (6:30–8:30PM), consuming a similar diet. Matched for exercise dose, using a multimodal training approach (Resistance, Interval, Stretch, Endurance; RISE), the major findings of the current study were: 1) in the women cohort, those who exercised in the AM had greater reductions in total (−5% vs −2%) and abdominal fat mass (−10% vs −3%) and blood pressure (−10% vs −3%), as well as increased lower body muscle power (13% vs 4%); whereas, women exercising in the PM had significantly greater gains in upper body muscle strength (16% vs 9%), power (37% vs 8%), and endurance (40% vs 25%), and a tendency to experience improved mood and greater satiety; and 2) in the men cohort, those exercise training in the PM experienced a significantly greater reduction in systolic blood pressure (−12% vs −3%) and feelings of fatigue (−55% vs 0%), and increased fat oxidation (6% vs 1%) compared to AM exercisers.

Collectively, these findings provide support for exercise-trained women to perform a multi-model exercise training regimen (RISE) in the morning to optimize total body and abdominal fat loss, lowering of blood pressure, and increasing lower body muscular power, whereas exercise in the evening may provide improvements in upper body muscular performance, and possibly mood enhancement. For exercise-trained men, multi-modal evening exercise may be more advantageous to reduce blood pressure and fatigue, as well as to maximally stimulate fat oxidation. Thus, women and men respond differently to ETOD and therefore, careful attention should be used in matching physical performance, cardiometabolic, and psychological mood state goals with the scheduling of multi-modal exercise training to optimize results.

### Impact of ETOD on Training-Induced Changes in Physical Performance

The major finding of the current study showed that, women engaged in the 12-week comprehensive multi-modal RISE exercise training program significantly improved all fitness-related performance outcomes, regardless of ETOD. However, women performing exercise in the evening experienced significantly greater training-induced increases in bench press maximal strength, upper body peak power, and maximal number of pushups. Interestingly, squat jump peak power was the one exception, which showed significantly greater improvement in the morning versus evening exercise groups, likely due to a slightly (non-significant) lower baseline starting level in the AM group. In contrast, these outcomes were less time divergent in men, with performance parameters significantly improved in both AM and PM exercisers These findings suggest that the time of day exercise is performed in women impacts physical performance outcomes to a greater degree than when men perform training, in partial agreement with previous literature (Seo et al., 2013; Basti et al., 2021). The precise mechanisms of this potential sexual dimorphism in ETOD effect on performance needs further study.

Previous research suggests that aerobic training and performance is less affected by time of day; whereas resistance training exhibits a clear trend towards greater benefits when performed in the afternoon/evening (Seo et al., 2013; Basti et al., 2021). The physiological underpinnings for the propensity for greater muscle performance in the afternoon/evening are likely complex and varied (Ammar et al., 2017). Specifically, increased core temperature, and alterations in redox balance, inflammatory profile, muscle metabolism and estimates of muscle remodeling (i.e., lactate, lactate dehydrogenase, creatine kinase), and muscle tone all show a circadian diurnal variation, at rest and in response to exercise (Ammar et al., 2017; Basti et al., 2021). Although some of these biochemical parameters exude a chronobiological pattern that may seem disadvantageous (e.g., increased oxidative stress, inflammation, etc.), others may prove beneficial such as increased core temperature and circulating lactate, which may act to increase nerve conduction velocity, metabolic rate (Ammar et al., 2017) and increase bioenergetic capacity (Brooks et al., 2021). Interestingly, recent work on the acute effects of exercise at different times of day (circadian rhythms), suggests molecular clock gene expression varies throughout the day which was correlated with muscular strength (Basti et al., 2021). Collectively, molecular, endocrine, metabolic, and neuromuscular factors likely contribute to the diurnal variation in muscle strength, which warrant continued investigation in training interventions.

### Impact of ETOD on Training-Induced Changes in Body Composition

In the present study, we report women performing multi-modal RISE training, regardless of time of day, drastically reduced total and regional body fat and increased percentage of fat-free mass. Most compelling, women who exercised in the AM experienced significantly greater reductions in total fat mass and percentage of abdominal fat mass than those women exercising in the PM. Thus, women exercise training in the AM may reduce abdominal and total adiposity over training in the PM, whereas, women training in the evening may derive superior gains in muscle performance. Perhaps overnight fasting, as suggested previously to increase the relative utilization of fat during aerobic (Achten and Jeukendrup, 2004; Beckers et al., 2008), and resistance exercise (Frawley et al., 2018), repeated over time, may result in greater adaptation and loss of adipose tissue mass.

Morning exercise is increasingly recognized to benefit exercise adherence and weight management in overweight/obese individuals (Schumacher et al., 2020; Willis et al., 2020). In the current study, adherence to morning and evening exercise was equal between groups. In fact, the current study extends the high degree of compliance/adherence our laboratory has shown previously in overweight/obese women and men following the RISE exercise regimen, and now includes normal weight active women and men. The explanatory mechanisms contributing to greater body composition changes in women exercising in the morning with the RISE intervention remain elusive.

Similar to the current findings in women, the men following the RISE fitness regimen, significantly improved body composition, regardless of exercise time of day. Mechanisms for an absence of differences in body composition between men exercising in the morning versus evening remains unknown but may simply indicate that body composition perturbations are more difficult to achieve in exercise-trained men with less room for improvement, or may take longer to manifest. It’s important to highlight that all participants were given meal plans to follow over the course of the study, and diet did not differ between groups over the course of the study, only time of day in which exercise was performed varied.

### Impact of ETOD on Training-Induced Changes in Cardiometabolic Outcomes

In the present study, women had significant reductions in systolic and diastolic blood pressures and aortic and brachial artery stiffness, with greater reductions in blood pressure occurring in the morning exercise group, likely attributed to higher baseline blood pressures in this group. The lower measures of vascular stiffness, now considered prognostic indicators of vascular health (Asmar et al., 1995; Willum Hansen et al., 2006), may be contributing to reductions in blood pressure. Although greater training-induced improvements in endothelial health, blood flow and vascular resistance (Kobayashi et al., 2004) may also be contributing (Aoyama and Shibata, 2020). Thus, women with borderline hypertension aiming to optimize blood pressure may consider exercise training in the morning.

In contrast, only men who exercised in the evening showed a reduction in systolic blood pressure. This was likely due to the same reason in the women, the evening exercises were borderline hypertensive and therefore had room for improvement. Mechanistically, it is unclear what factors are contributing to this greater hypotensive effect of AM or PM exercise, but may be related to exercise being performed after the nocturnal dipping (Palatini et al., 2020) or might blunt the “morning surge” in blood pressure, but may also depend upon nocturnal dipping status (Aoyama and Shibata, 2020). In a randomized crossover study of pre-hypertensive men, the post-exercise hypotensive effect was greater following exercise in the morning rather than the evening (de Brito et al., 2015). Thus, in the current cohort of women with slightly elevated blood pressure in the AM group, a healthy nocturnal dipping status would be expected to follow the greater hypotensive effect of morning exercise (de Brito et al., 2015; Aoyama and Shibata, 2020). The mechanisms for the reduction in systolic blood pressure in men following evening exercise is to be determined but may be related to circadian interactions of evening exercise and hormonal factors.

Additional improvements in cardiometabolic parameters included reductions of total cholesterol in both AM and PM women exercisers and total cholesterol to high-density lipoprotein cholesterol ratio reduction observed only in the evening men exercisers. The congruency of reductions in blood pressure and blood lipids in response to multi-modal exercise training in both women and men is encouraging and reinforces the necessity of physical exercise to optimize cardiovascular health. Other cardiometabolic health biomarkers, including circulating levels of lipids, glucose, insulin, high sensitivity C-reactive protein, and inflammation (IL-6, cortisol) were not significantly impacted by ETOD in both women and men, as most of these variables were within low to normal ranges.

The current study observed several intriguing metabolic findings. Namely, significant reductions in resting metabolic rate (RMR) in AM exercising women and respiratory exchange ratio (RER) in PM exercising men compared to their exercise time of day counterparts. The reduced RMR is not an uncommon finding in highly trained subjects (Woods et al., 2018), and may be related to both enhanced metabolic efficiency at rest and/or reduced energy availability (Sterringer and Larson-Meyer, 2022). Indeed, the women in the current study self-reported only ∼1650 kcals/day during the 12-week exercise intervention and may have created a cascade of neuro-hormonal perturbations resulting in a state of metabolic conservation. While a fasting-induced enhanced fat utilization hypothesis may partly explain the greater reduction in total and abdominal fat loss in the AM group of women, the significant drop in RMR complicates fully accepting this mechanism. Moreover, our study design does not rule out a possible difference may exist in energy expenditure flux during exercise, as documented previously (Achten and Jeukendrup, 2004; Frawley et al., 2018), and the relative substrate utilization during exercise was not assessed over the intervention in the present study and thus cannot be ascertained.

Our finding of a significant increase in fat oxidation (lower RER) in men exercising in the evening is likely related to circadian regulation of metabolism and the well-accepted finding that metabolism is highest in the late afternoon/early evening (Zitting et al., 2018). The addition of performing exercise training during this time of day may provide a “metabolic advantage” in terms of enhanced body fat mass loss, although the current study found similar amounts of total and regional body fat loss in AM and PM exercisers. This suggests the metabolic benefit of increased fat oxidation precedes a possible latent effect of actual fat loss which may occur beyond the duration of the current study intervention (12 weeks), and argues for longer-term ETOD training interventions on circadian rhythm regulation of energy metabolism, body composition, and cardiometabolic health outcomes.

### Impact of Exercise Time of Day on Training-Induced Changes in Mood, Satiety, and Hunger

A unique component of the current study was the inclusion of perceived mood and hunger/satiety changes to determine whether relationships exist with how the body responds and adapts to exercise time of day (AM versus PM) and the biological clock. Our findings show women experience relatively small mood enhancement benefit from exercise (only PM exercisers reduced tension and total mood disturbance (TMD)). However, men demonstrate significant mood improvement to both morning and evening exercise, including reduced tension, depression, anger, fatigue, confusion, and total mood disturbance (TMD), and increased vigor, with evening exercisers experiencing considerably less fatigue. These findings provide compelling novel data regarding circadian clock differences in mood state regulation based on exercise time of day between women and men. Thus, women appear less responsive to the mood “boosting” effects of exercise compared to men, regardless of when exercise is performed during the day.

Alternatively, another possible explanation for the heightened impact of circadian rhythmicity and training time of day upon changes in adiposity may be related to variations in feelings of satiety and hunger. Physiologically, changes in circulating hormones such as leptin, ghrelin, peptide YY, etc., aside from eliciting direct metabolic effects, are also known to impact psychological sensations of hunger or satiety (Austin and Marks, 2009). Though such hunger/satiety hormones were not assessed in the present study, visual analog scale-based assessments of hunger (“how hungry are you” & “how much food could you eat”) and satiety (“how full are you?”) were performed and revealed only minimal differences in women over the intervention. Collectively, these results suggest exercising in the AM, while more favorable for reducing total and abdominal fat loss in women, does not seem to be explained by perceptions of satiety or hunger. Thus, mood and hunger/satiety perceptions may work in tandem as they impact body composition.

Teleologically, training-induced changes in satiety-hunger hormones, differentially impacted by ETOD could contribute to alterations in energy intake and thus adiposity or directly through impacts on fat metabolism (Austin and Marks, 2009). However, previous work in obese women suggests training does not alter adipokine expression and actually decreases circulating leptin (Polak et al., 2006), as suggested elsewhere (Bouassida et al., 2010). Directly measuring food intake and satiety-hunger regulating hormones may help exclude these as potential mechanisms underpinning the superior body compositional adaptations as a result of AM training and/or physical performance outcomes due to PM exercise in women. Further work is needed to discern the impact of training time of day and its potential impact on circadian regulation of diet intake, metabolism, and hunger-satiety.

There are several limitations of the current study worth mentioning. First, we included only exercise-trained men and women and therefore our results may not be applicable to less-trained and/or overweight women and men. It is important to highlight the inclusion of a wide variety of well-conditioned subjects ranging from anaerobic, aerobic, strength, power, and team sport athletes to demonstrate efficacy from our findings. Second, our volunteer and subsequent participant cohort were mostly Caucasian women and men and thus under-represents minority groups. Third, the focus of this study was to comprehensively investigate the clinical and/or functional adaptations, in a relatively non-invasive manner, detailed molecular mechanisms were not possible. Thus, we did not measure markers of redox balance, an expansive inflammatory profile, and several pertinent factors involved in muscle metabolism and remodeling (i.e., lactate, lactate dehydrogenase, creatine kinase, tec.) that are likely to be involved with and/or underpin many of the outcomes assessed in our investigation. However, based on the novelty of this work and the already wide array of measures, our data provides a strong basis for future ETOD research to pursue these outcomes. Fourth, we did not determine chronotype or ETOD preference, but randomly assigned participants to ETOD, while a scientifically valid approach, it begs the question if exercise prescription based on chronotype (AM vs PM) could possibly result in even greater adaptations. On a related note, this study occurred during late January—early April, and therefore included a month of daylight savings time which may have influenced the outcomes. Fifth, we did not include a mid-day group in our study design, instead focusing on early AM and later PM periods. While mid-day training warrants investigation in the context of ETOD research, our work considered real-life applicability with the adult population enrolled in this study. Finally, we did not include a no-exercise group which would have provided better contextualization of many of the performance and cardiometabolic outcomes in the present analysis with known diurnal variation (i.e., magnitude of adaptations for AM training (vs AM-tested control) compared to the magnitude of adaptations for PM training (vs PM-tested control)). Indeed, when reported in this manner, a recent meta-analysis found strength differences between AM and PM training were non-significant (Grgic et al., 2019). However, the majority of participants that were pooled for these meta-analyses were not exercise-trained individuals and may have benefit from exercise in general, regardless of ETOD. While future work should seek to include this “control” element by having participants abstain from exercise, we note recruitment may be difficult in trained individuals.

## Conclusion

In conclusion, the time of day when multi-modal (resistance, intervals, stretching, endurance; RISE) exercise training is performed (AM versus PM) has profound impacts on cardiometabolic, body composition, and physical performance outcomes, which appear to differentially manifest in women and men. Morning exercise in women, enhances total and abdominal fat loss, reduces blood pressure, and increases lower body muscle power, whereas, evening exercise greatly increases upper body muscle strength, power, and endurance, and enhances overall mood. For men, evening exercise lowers systolic blood pressure and fatigue, and stimulates fat oxidation compared to early morning exercise. These findings highlight the interaction of exercise time of day (AM versus PM) and circadian regulation and the impact this has on cardiometabolic, body composition, and physical performance outcomes in healthy, exercise-trained women and men following twelve weeks of multi-modal training. As such, the length of training may also influence sex differences. Healthcare clinicians and fitness trainers/practitioners aiming to more precisely target or emphasize one outcome over the other should bear in mind time of day when making exercise and physical activity recommendations to individual patients or clients.

## Data Availability

The raw data supporting the conclusion of this article will be made available by the authors, without undue reservation.
